# Global burden of plastic-surgery-related conditions, 1990–2021: a composition-aware analysis with projections to 2050

**DOI:** 10.3389/fpubh.2025.1676386

**Published:** 2025-11-12

**Authors:** Chengcheng Zhang, Xi Chen, Wenjuan Ying, Chaoqun Zhang

**Affiliations:** 1 The First Affiliated Hospital of Shantou University Medical College, Shantou, Guangdong, China; 2 Shantou University Medical College, Shantou, Guangdong, China; 3 The First Affiliated Hospital of Xinjiang Medical University, Urumqi, China; 4 The 7th People's Hospital of Zhengzhou, Zhengzhou, China

**Keywords:** plastic surgery–related diseases, global burden, age-standardized rates, Bayesian age-period-cohort model, disease projection, SDI disparities

## Abstract

**Background:**

Plastic surgery–related diseases impose a substantial and growing burden on global health systems, yet comprehensive estimates of their temporal trends and future projections remain scarce. This study aims to quantify the global, regional, and national burden of 12 plastic surgery–relevant conditions from 1990 to 2021 and to forecast their trajectories through 2050.

**Method:**

Data were extracted from the Global Burden of Disease Study 2021. We analyzed age-standardized incidence rate (ASIR), prevalence rate (ASPR), death rate (ASDR), and disability-adjusted life years (DALYs) across 204 countries and territories. Estimated annual percentage changes (EAPCs) were calculated to evaluate temporal trends, and a Bayesian age-period-cohort (BAPC) model was employed to project disease burden to 2050. Socio-demographic disparities were assessed using SDI-based stratification and Spearman correlation.

**Findings:**

Globally, incidence was compositionally dominated by pyoderma (about 90% in 1990–2021). Incident cases increased from 508.18 million (95% UI 489.85–527.22) in 1990 to 902.35 million (95% UI 871.47–934.10) in 2021, with an ASIR increasing from 9,611.94 to 11,560.66 per 100,000 population. In contrast, the ASPR declined from 2,862.67 to 2,207.26 per 100,000, and age-standardized mortality and DALYs rates also fell. The burden rose steeply with age: adults aged ≥60 years consistently exhibited the highest age-specific rates, and the absolute burden progressively shifted toward older populations. High-SDI regions had lower age-standardized mortality and DALYs rates than low-SDI regions, despite occasionally higher prevalence consistent with improved survival. By 2050, the absolute burden is projected to grow with population aging, while most age-standardized rates continue to decline.

**Interpretation:**

Aggregate incidence increases were driven mainly by pyoderma. Surgically relevant burdens concentrated in oncologic and burn/trauma cohorts; hence service implications should be anchored to these metrics rather than incidence. By 2050, incidence continues to rise largely from infections, while DALYs trajectories in surgically managed subgroups diverge by SDI-supporting targeted prevention, context-specific capacity strengthening, and integrated rehabilitation.

## Introduction

Plastic surgery–related diseases encompass a broad spectrum of conditions—including cancers, congenital anomalies, soft tissue infections, ulcers, and trauma—that frequently require surgical intervention, wound reconstruction, or aesthetic rehabilitation (1, 2). These conditions not only cause significant morbidity and mortality but also impose considerable social and economic burdens through disfigurement, disability, and long-term care needs (3, 4). In particular, diseases such as breast cancer, orofacial clefts, skin malignancies, and burns disproportionately affect vulnerable populations and often necessitate complex surgical management across both acute and chronic care settings (5, 6).

Despite their clinical and public health relevance, plastic surgery–related conditions have not been adequately captured in global health surveillance frameworks (1, 7). Existing research often focuses on individual diseases or narrow geographic regions, limiting cross-country comparability (8). Moreover, many studies rely on hospital-based data, which underrepresents populations in low- and middle-income settings where access to surgical care remains limited (9). There is a notable lack of comprehensive, standardized estimates that quantify long-term trends in incidence, prevalence, mortality, and disability burden across the full spectrum of diseases relevant to plastic surgery (4). Additionally, few studies have attempted to forecast future burden using robust demographic models, hindering efforts to align surgical system strengthening with projected needs (10).

In this context, we conducted a global, regional, and national assessment of 12 plastic surgery–related diseases using data from the Global Burden of Disease Study (GBD) 2021 (11). We recognize the substantial heterogeneity among these conditions—spanning high-incidence integumentary infections and low-incidence, high-complexity reconstructive diseases—which presents inherent challenges for aggregated analysis. This compositional diversity may disproportionately influence overall metrics; hence, we deliberately differentiate between infection-driven incidence and surgery-relevant disability burdens throughout our analysis. Our study aims to quantify historical trends in burden from 1990 to 2021, evaluate disparities across sociodemographic strata, and project disease trajectories through 2050 using a Bayesian age–period–cohort (BAPC) model. By generating robust, comparable, and policy-relevant estimates, this study addresses a critical gap in global health research and provides evidence to guide investment in surgical capacity, resource allocation, and disease prevention strategies in the decades ahead.

## Methods

### Data sources and case definition

This study utilized publicly available data from the GBD 2021, developed by the Institute for Health Metrics and Evaluation (11). The GBD 2021 provides comprehensive, comparable, and annually updated estimates of incidence, prevalence, mortality, years of life lost (YLLs), years lived with disability (YLDs), and disability-adjusted life years (DALYs) for 369 diseases and injuries across 204 countries and territories from 1990 to 2021. Data sources integrated into the GBD framework include national health surveys, disease registries, hospital discharge records, claims data, vital registration systems, and scientific literature. The GBD employs standardized estimation methods to adjust for underreporting, misclassification, and sparse data, including Bayesian meta-regression modeling (DisMod-MR 2.1), spatiotemporal Gaussian process regression, and ensemble modeling (12). All data were obtained through the Global Health Data Exchange (GHDx: https://ghdx.healthdata.org/).

Twelve conditions were analyzed as plastic-surgery–related: they are relevant to reconstructive care but do not uniformly require surgery—cancers, orofacial clefts, and thermal burns frequently require reconstruction, whereas many infections (e.g., pyoderma) are primarily managed in primary care. Composite totals are presented descriptively; surgical inferences are anchored to deaths and DALYs. To minimize subjectivity and ensure reproducibility, selection followed a prespecified framework: (i) alignment with GBD 2021 nosology and explicit ICD-9/10 mapping (Level 3–4 causes); (ii) routine relevance to reconstructive pathways; (iii) non-trivial DALY burden in GBD 2021; and (iv) time-series completeness (1990–2021). We applied rule-based exclusions where GBD coding lacks granularity to isolate plastic-surgery–specific caseloads (e.g., some upper-extremity trauma subsets), consistent with prior specialty-focused GBD work (13–16).

The final set comprised breast cancer; malignant melanoma; non-melanoma skin cancer (keratinocyte carcinomas, i.e., basal-cell and squamous-cell combined); lip and oral cavity, nasopharynx, other pharynx, and larynx cancers; orofacial clefts; thermal burns; cellulitis; and pyoderma. Accounting follows GBD 2021 Level-3 categories—unlike some national analyses that list BCC and SCC separately, GBD aggregates these as non-melanoma skin cancer to preserve global comparability. ICD-9/10 ranges follow the official GBD non-fatal crosswalk (including hospital/claims variants).

### Socio-demographic index

To evaluate disparities across development levels, all countries were stratified into five quintiles based on the Socio-demographic Index (SDI), a composite metric of income per capita, mean years of education among those aged 15 and older, and total fertility rate under age 25 (11). SDI ranges from 0 (lowest development) to 1 (highest development) and allows comparison across low, low-middle, middle, high-middle, and high SDI groups. Trends in burden indicators were examined in relation to SDI values to assess socioeconomic gradients and global inequality.

### Bayesian Age–Period–Cohort model

The BAPC model was employed to assess temporal trends and forecast the future burden of plastic surgery–related diseases through 2050. This model decomposes the disease burden into three temporal dimensions and is mathematically expressed as:


$$\begin{array}{l} Y_{ijk}=\mu +\alpha_{i}+\beta_{j}+\gamma_{k}+\epsilon_{ijk} \end{array}$$

where *Y*_*ijk*_ represents the observed disease burden for age group *i*, period *j*, and cohort *k*; is the overall intercept; and ϵ_*ijk*_ is the random error term.

Age effect (α_*i*_): captures age-related differences in disease risk, reflecting biological and demographic influences.

Period effect (β_*j*_): represents time-specific influences that impact all age groups equally, such as healthcare advances, disease surveillance, or policy changes.

Cohort effect (γ_*k*_): accounts for generational variation in exposure to risk factors based on year of birth.

Parameter estimation was conducted using the INLA algorithm within the BAPC package in R. This method enables efficient Bayesian inference without reliance on Markov Chain Monte Carlo. Posterior distributions and 95% credible intervals were generated for all model parameters. Because the BAPC model extrapolates from temporal patterns, fitted values for baseline years (e.g., 1990, 2021) may not exactly reproduce the observed GBD estimates; such minor discrepancies reflect model structure rather than analytical error. Model adequacy and convergence were assessed using posterior predictive checks and diagnostic plots, ensuring the robustness and reliability of long-term forecasts (17).

### Statistical analysis

We calculated age-standardized rates (ASRs) per 100,000 population using the direct method, adjusting for population age structure based on the GBD global standard population (11). We report age-standardized incidence (ASIR), prevalence (ASPR), death (ASDR), and DALY rates. The ASR was derived through a weighted average of age-specific rates, computed as:


$$\begin{array}{l} ASR=\frac{\overset{N}{\underset{i=1}{\sum }}a_{i}w_{i}}{\overset{N}{\underset{i=\;1}{\sum }}w_{i}} \end{array}$$

Where *a*_*i*_ is the age-specific rate in the *i*^*th*^ age group, *w*_*i*_ is the population weight for the same age group in the standard population, and *N* is the total number of age groups.

To quantify temporal changes in ASR, we estimated the estimated annual percentage change (EAPC) using a log-linear regression model fitted to annual ASR values:


$$\begin{array}{l} \text{ln}\left(\text{ASR}\right)\;=\text{ α }+\text{ β }\times \text{ year }+\text{ ε} \end{array}$$

in which α and β represent the intercept and slope, respectively, and ε denotes the residual error. The EAPC was calculated by:


$$\begin{array}{l} \text{EAPC }=\;100\times \left(\text{exp}\left(\text{β}\right)\;-\;1\right) \end{array}$$

with 95% confidence intervals (CIs) derived from the regression model (18). A statistically meaningful trend was defined as one with a 95% CI that does not encompass zero.

To investigate the association between ASRs and national development status, we applied penalized spline regression to examine potential non-linear relationships with the SDI. Additionally, Spearman's rank correlation was used to assess monotonic associations, with significance defined at a two-tailed *P*-value less than 0.05.

Detailed methods, diagnostics, and sensitivity analyses are provided in Supplementary methods S1–S10. All statistical analyses and graphical outputs were performed using R software (version 4.2.2). Key packages used included ggplot2 for data visualization, mgcv for spline modeling, and BAPC and INLA for projection analyses.

## Results

### Global burden of plastic surgery-related diseases

Globally, ASIR was dominated by pyoderma, accounting for 89.78% in 1990 and 91.84% in 2021. This predominance provides the context for interpreting subsequent incidence patterns. Incident cases increased from 508.18 million (95% UI 489.85–527.22) in 1990 to 902.35 million (871.47–934.10) in 2021 (Table 1), with ASIR rising from 9,611.94 (9,281.93–9,964.56) to 11,560.66 (11,171.01–11,968.75) per 100,000 [EAPC 0.61 (95% CI 0.60–0.63)]. Prevalent cases also grew, from 135.62 million (119.07–155.59) to 182.54 million (161.20–207.33), whereas ASPR declined from 2,862.67 (2,503.70–3,285.04) to 2,207.26 (1,952.83–2,501.88) per 100,000 [EAPC −0.87 (−0.91 to −0.83); Supplementary Table S1]. Deaths rose from 0.90 million (0.81–0.97) to 1.52 million (1.37–1.66), yet ASDR fell from 22.08 (20.04–23.80) to 17.99 (16.15–19.60) per 100,000 [EAPC −0.78 (−0.83 to −0.73); Supplementary Table S2]. Total DALYs increased from 36.59 million (32.27–40.91) to 49.83 million (44.66–55.08), while the age-standardized DALYs rate decreased from 789.09 (704.41–874.30) to 590.35 (526.32–654.29) per 100,000 [EAPC −1.06 (−1.10 to −1.01); Supplementary Table S3].

**Table 1:** The number of incidence cases and the ASIR of plastic surgery in 1990 and 2021, and its temporal trends from 1990 to 2021.

**Characteristic**	**1990**		**2021**		**1990–2021**
	**Cases (million, 95% UI)**	**ASIR/100,000 (95% UI)**	**Cases (million, 95% UI)**	**ASIR/100,000 (95% UI)**	**EAPC (95%CI)**
Global	508.18 (489.85, 527.22)	9,611.94 (9,281.93, 9,964.56)	902.35 (871.47, 934.10)	11,560.66 (11,171.01, 11,968.75)	0.61 (0.60, 0.63)
**SDI level**					
High SDI	61.83 (59.15, 64.69)	6,852.07 (6,567.84, 7,153.72)	83.46 (79.95, 87.12)	6,909.37 (6,629.14, 7,198.84)	0.05 (0.00, 0.10)
High-middle SDI	67.29 (64.60, 69.93)	6,427.39 (6,180.80, 6,674.64)	83.15 (79.98, 86.31)	6,233.22 (5,992.58, 6,477.21)	−0.10 (−0.11, −0.09)
Middle SDI	102.21 (98.29, 106.34)	6,158.29 (5,940.87, 6,385.82)	180.77 (174.31, 187.28)	7,422.59 (7,170.74, 7,688.01)	0.62 (0.60, 0.64)
Low-middle SDI	165.77 (160.22, 171.73)	14,793.04 (14,327.01, 15,301.98)	301.21 (291.53, 311.51)	16,046.68 (15,546.40, 16,584.16)	0.27 (0.25, 0.28)
Low SDI	110.78 (106.92, 114.74)	22,279.24 (21,544.46, 23,031.48)	253.33 (244.55, 262.38)	23,001.84 (22,247.79, 23,778.71)	0.11 (0.10, 0.12)
**Regions**					
Andean Latin America	1.79 (1.70, 1.89)	5,045.96 (4,826.92, 5,272.70)	3.24 (3.09, 3.39)	5,040.32 (4,812.40, 5,272.57)	−0.01 (−0.01, −0.01)
Australasia	3.64 (3.48, 3.80)	17,763.92 (17,017.62, 18,507.78)	5.84 (5.60, 6.08)	17,843.16 (17,098.16, 18,579.84)	0.04 (0.03, 0.05)
Caribbean	1.79 (1.70, 1.88)	5,393.66 (5,130.91, 5,652.98)	2.63 (2.51, 2.75)	5,439.07 (5,185.68, 5,696.75)	0.03 (0.02, 0.05)
Central Asia	5.20 (4.95, 5.44)	7,570.86 (7,230.38, 7,907.07)	7.24 (6.92, 7.56)	7,692.50 (7,364.71, 8,012.00)	0.07 (0.04, 0.09)
Central Europe	7.92 (7.58, 8.27)	6,236.62 (5,971.54, 6,512.17)	7.98 (7.66, 8.32)	6,142.97 (5,885.09, 6,397.93)	−0.06 (−0.07, −0.05)
Central Latin America	6.62 (6.18, 7.11)	4,316.16 (4,072.04, 4,586.21)	10.54 (10.02, 11.13)	4,212.26 (4,005.58, 4,447.78)	0.02 (−0.02, 0.06)
Central Sub-Saharan Africa	14.79 (14.15, 15.50)	27,277.54 (26,226.90, 28,436.28)	37.01 (35.36, 38.54)	27,720.43 (26,617.62, 28,798.95)	0.08 (0.07, 0.10)
East Asia	32.09 (30.52, 33.63)	2,771.42 (2,648.38, 2,897.56)	41.27 (39.41, 43.23)	2,695.57 (2,575.62, 2,817.27)	−0.17 (−0.21, −0.13)
Eastern Europe	27.10 (26.11, 28.12)	11,771.62 (11,358.66, 12,199.64)	26.42 (25.50, 27.41)	11,905.26 (11,487.54, 12,335.59)	0.05 (0.04, 0.07)
Eastern Sub-Saharan Africa	50.87 (49.03, 52.75)	25,973.56 (25,132.05, 26,875.72)	113.30 (109.19, 117.45)	26,333.89 (25,493.73, 27,208.47)	0.04 (0.02, 0.05)
High-income Asia Pacific	6.18 (5.86, 6.49)	3,753.40 (3,566.76, 3,935.29)	6.52 (6.22, 6.82)	3,633.11 (3,463.90, 3,800.68)	−0.13 (−0.14, −0.12)
High-income North America	15.97 (14.93, 17.07)	5,450.68 (5,113.89, 5,809.77)	25.23 (23.73, 26.77)	5,687.61 (5,372.24, 6,016.39)	0.34 (0.21, 0.47)
North Africa and Middle East	31.90 (30.75, 33.09)	9,237.42 (8,896.06, 9,575.26)	57.03 (54.96, 59.10)	9,264.22 (8,927.45, 9,579.79)	0.03 (0.02, 0.03)
Oceania	0.22 (0.21, 0.23)	3,694.19 (3,534.68, 3,853.93)	0.50 (0.48, 0.52)	3,926.18 (3,761.50, 4,101.05)	0.20 (0.20, 0.21)
South Asia	183.44 (177.38, 190.03)	17,646.79 (17,090.08, 18,248.50)	334.54 (323.43, 346.28)	18,635.62 (18,048.95, 19,282.33)	0.17 (0.16, 0.18)
Southeast Asia	7.10 (6.71, 7.54)	1,736.59 (1,648.80, 1,825.88)	11.31 (10.71, 11.91)	1,679.88 (1,595.66, 1,765.30)	−0.13 (−0.17, −0.08)
Southern Latin America	5.43 (5.17, 5.67)	11,028.10 (10,523.48, 11,522.82)	7.82 (7.48, 8.15)	11,251.68 (10,754.52, 11,733.01)	0.08 (0.07, 0.10)
Southern Sub-Saharan Africa	13.96 (13.50, 14.46)	27,287.50 (26,410.97, 28,195.92)	21.58 (20.82, 22.35)	27,603.59 (26,681.24, 28,531.13)	0.04 (0.03, 0.05)
Tropical Latin America	7.11 (6.77, 7.46)	5,267.17 (5,039.55, 5,502.43)	12.76 (12.25, 13.30)	5,419.13 (5,209.22, 5,643.14)	0.03 (−0.02, 0.08)
Western Europe	34.85 (33.58, 36.22)	8,624.97 (8,300.93, 8,955.48)	41.55 (40.02, 43.10)	8,658.68 (8,339.46, 8,978.86)	−0.02 (−0.04, −0.00)
Western Sub-Saharan Africa	50.22 (48.59, 51.96)	26,758.84 (25,922.85, 27,592.81)	128.06 (123.93, 132.52)	27,259.89 (26,428.50, 28,136.06)	0.10 (0.08, 0.13)

ASIR, age-standardized incidence rate; EAPC, estimated annual percentage change.

### Regional and national burden of plastic surgery-related diseases

In 2021, incident cases were highest in South Asia (334.54 million; 95% UI 323.43–346.28), Western Sub-Saharan Africa (128.06 million; 123.93–132.52), and Southeast Asia (113.09 million; 107.13–119.12; Table 1). The highest ASIR occurred in Southern Sub-Saharan Africa (27,603.59 per 100,000; 26,681.24–28,531.13), followed by Western Sub-Saharan Africa (27,259.89; 26,428.50–28,136.06). ASIR increased in most regions, with the largest rise in High-income North America (EAPC 0.34; 0.21–0.47). Prevalent cases were most numerous in South Asia (28.29 million; 25.52–31.43), East Asia (20.53 million; 17.07–24.73), and High-income North America (17.50 million; 15.11–20.13), while the steepest decline in ASPR occurred in Tropical Latin America [from 3,299.55 to 2,138.21; EAPC −2.05 (−2.27 to −1.82); Supplementary Table S1].

In terms of mortality, South Asia (0.37 million; 0.31–0.43) and East Asia (0.22; 0.18–0.28) had the largest numbers of deaths (Supplementary Table S2). ASDR declined in most settings—particularly East Asia [from 17.13 to 10.66; EAPC −1.79 (−1.89 to −1.69)], Western Europe [from 27.40 to 17.76; −1.42 (−1.47 to −1.37)], and High-income North America [from 25.86 to 16.55; −1.46 (−1.51 to −1.42)]—but increased in Western Sub-Saharan Africa [0.73 (0.69–0.78)] and Southern Sub-Saharan Africa [0.58 (0.35–0.82)]. DALYs were highest in South Asia (13.01 million; 10.95–15.03) and East Asia (6.88 million; 5.43–8.59; Supplementary Table S3). The age-standardized DALYs rate fell in nearly all regions—most notably East Asia [from 611.38 to 332.21; −2.28 (−2.42 to −2.13)] and Central Asia [from 1,034.82 to 568.18; −2.15 (−2.30 to −2.01)]—with Western Sub-Saharan Africa the only region showing an increase [from 635.72 to 692.27; 0.30 (0.24–0.36)].

**Figure 1:**
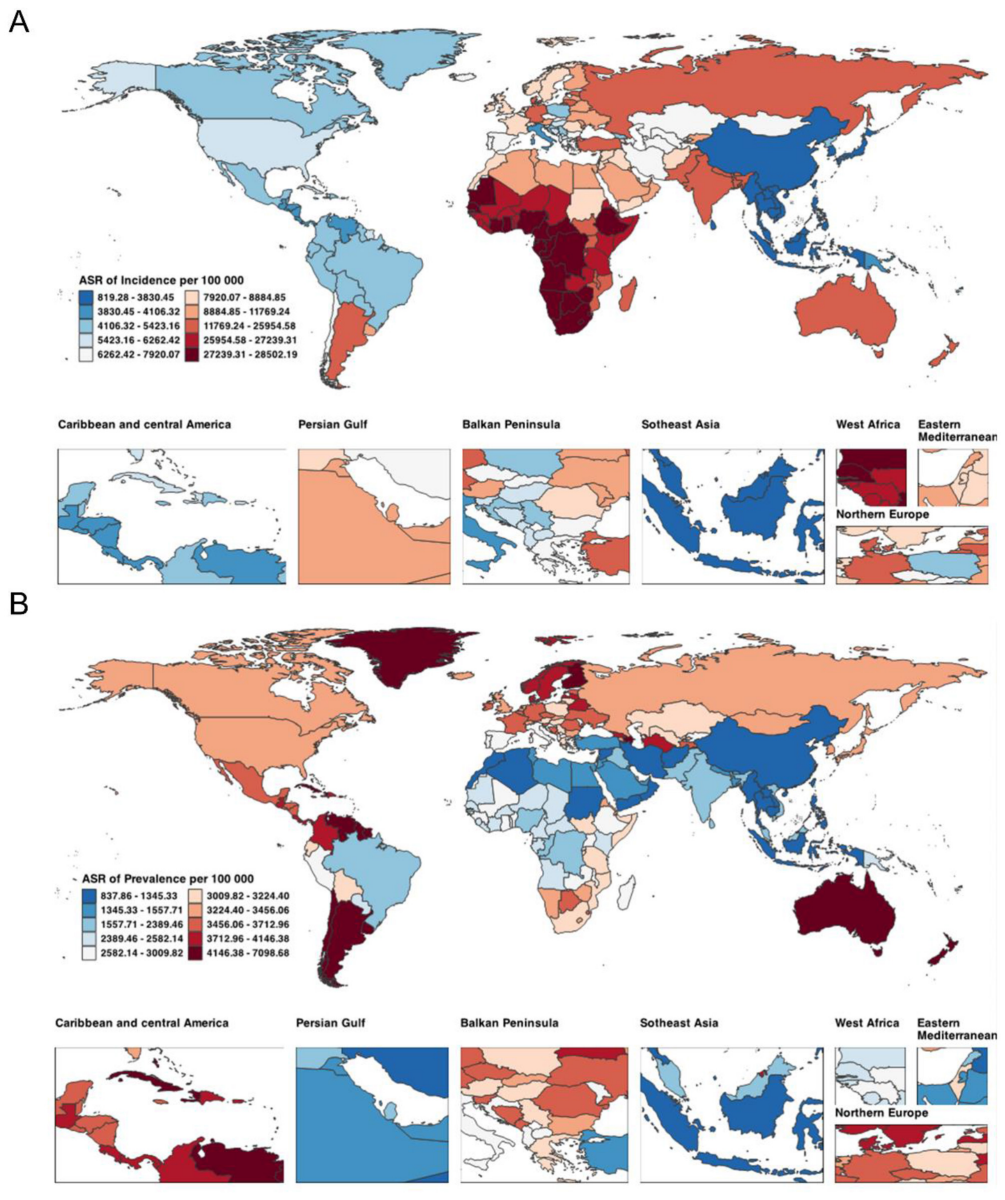
Global distribution of ASIR **(A)** and ASPR **(B)** attributable to Plastic Surgery-Related Diseases in 2021. ASIR, age-standardized incidence rate; ASPR, age-standardized prevalence rate.

In 2021, the largest numbers of incident cases were recorded in India (257.14 million; 95% UI 248.61–266.12) and China (37.75 million; 35.98–39.60). The highest ASIR was observed in Equatorial Guinea [27,207.72 in 1990 rising to 28,219.99 per 100,000 in 2021; EAPC 0.16 (0.14–0.17)], with Gabon and Eswatini also among the countries with sustained increases over three decades (Figure 1A). Prevalent cases were concentrated in Bolivia (Plurinational State of) (22.39 million; 20.14–24.98), Turkmenistan (19.84 million; 16.43–23.96), and Estonia (15.79 million; 13.65–18.16). The largest ASPR reductions were seen in Bahrain [from 1,493.67 to 1,346.21 per 100,000; EAPC −8.89 (−11.73 to −5.96)] and Egypt [from 4,676.22 to 2,112.75; −2.79 (−3.02 to −2.55); Figure 1B]. In terms of mortality, China (0.21 million; 0.16–0.26), the USA (0.10 million; 0.08–0.10), and Pakistan (0.06 million; 0.04–0.08) had the highest numbers of deaths in 2021. Most countries experienced declining ASDR, with the greatest reduction in Greenland [from 51.43 to 24.46 per 100,000; EAPC −2.50 (−2.61 to −2.38); Supplementary Figure S1A]. The highest numbers of DALYs were observed in Albania (9.84 million; 8.10–11.55), Bahamas (8.56 million; 7.48–9.68), and Belgium (8.16 million; 6.98–9.36). Despite increases in absolute DALYs in many countries, the age-standardized DALYs rate declined in most settings—especially Palau, New Zealand, and the United States Virgin Islands—whereas Southern Sub-Saharan Africa showed slight upward trends (Supplementary Figure S1B).

### Age disparities in the global burden of plastic surgery-related diseases

**Figure 2:**
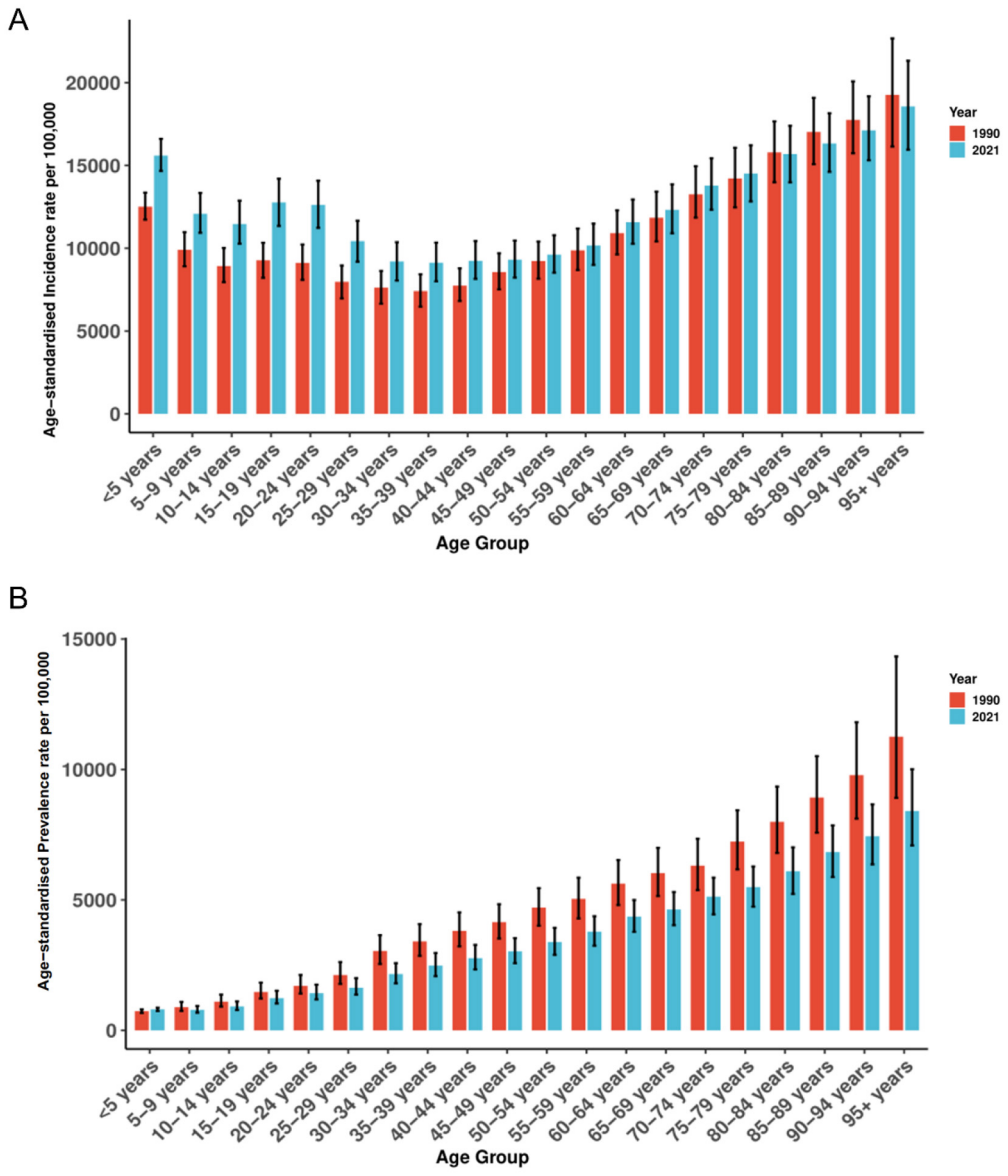
The distribution of ASIR **(A)** and ASPR **(B)** across Plastic Surgery-Related Diseases by age in 1990 and 2021. ASIR, age-standardized incidence rate; ASPR, age-standardized prevalence rate.

From 1990 to 2021, ASIR showed a pronounced U-shaped age profile (Figure 2A). In 2021 the highest rates occurred in those aged ≥95 years (18,563.49 per 100,000; 95% UI 15,953.39–21,326.20), followed by ages 90–94 (17,119.72; 15,313.92–19,176.44) and 85–89 years (16,330.18; 14,616.85–18,152.83); the lowest rate was in adults aged 35–39 years (9,124.77; 8,004.61–10,337.15). Relative to 1990, ASIR increased across almost all age groups in 2021, with the largest gains in children < 5 years [12,513.92 (11,730.14–13,356.68) to 15,598.71 (14,670.79–16,606.47)] and adolescents 15–19 years [9,271.50 (8,214.26–10,329.25) to 12,766.15 (11,348.91–14,197.61)]. Older age groups (≥80 years) remained consistently high across both years with only minor fluctuation.

In 2021, ASPR rose steadily with age (Figure 2B), peaking at ≥95 years (8,409.50 per 100,000; 95% UI 7,089.52–10,009.82), followed by 90–94 years (7,440.67; 6,362.56–8,659.19) and 85–89 years (6,833.93; 5,876.73–7,855.42); the lowest rate was in ages 5–9 years (786.90; 678.71–932.91). Compared with 1990, ASPR declined in nearly all age groups, with the steepest reductions at older ages-for example, 80–84 years decreased from 7,992.38 (6,801.87–9,343.77) to 6,097.25 (5,228.39–7,012.54). Despite these declines, a clear age gradient persisted from adolescence through older age.

From 1990 to 2021, mortality increased sharply with age (Supplementary Figure S2A). In 1990, ASDR ranged from 9.78 (95% UI 6.32–13.23) per 100,000 in children < 5 years to 416.25 (317.95–476.32) in those ≥95 years. In 2021, the lowest ASDR was in ages 10–14 years at 0.54 (0.39–0.66) and the highest again in ≥95 years at 449.37 (327.13–518.92). Most age groups recorded declines vs. 1990, notably ages 40–44 years [16.15 (14.77–17.55) to 13.29 (11.89–14.63)] and 55–59 years [51.16 (47.14–55.43) to 41.69 (37.68–45.80)].

DALYs burden in 2021 increased markedly with age (Supplementary Figure S2B), from a nadir in ages 10–14 years (67.51 per 100,000; 95% UI 52.17–83.88) to a peak in ≥95 years (3,888.93; 2,880.25–4,495.52); rates exceeded 1,000 per 100,000 from age 50 years upwards. Compared with 1990, DALYs rates declined across most ages-for example, < 5 years decreased from 882.32 (575.16–1,190.21) to 303.12 (168.42–449.93)-but increased in the oldest group [≥95 years: 3,706.4 (2,888.6–4,256.9) to 3,888.9 (2,880.3–4,495.5)], underscoring a persistent and growing burden in aging populations.

### Cause-specific composition of the global burden of plastic surgery–related diseases

**Figure 3:**
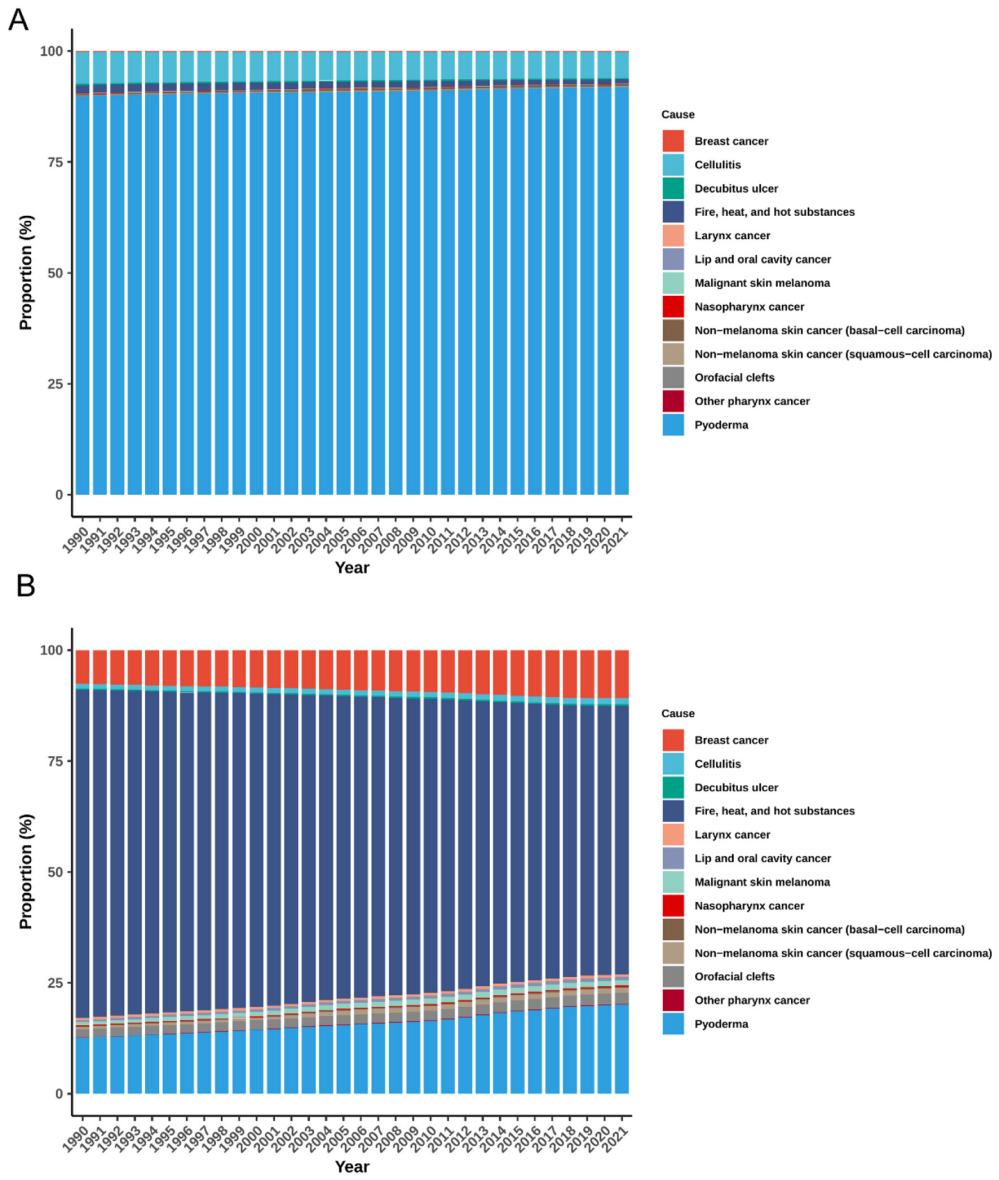
Temporal trends in cause-specific composition of ASIR **(A)** and ASPR **(B)** for global plastic surgery-related diseases, 1990–2021. ASIR, age-standardized incidence rate; ASPR, age-standardized prevalence rate.

Consistent with the opening summary, pyoderma dominated the composition throughout 1990–2021 (Figure 3A). Cellulitis and decubitus ulcer ranked second and third, maintaining proportions of approximately 5.87%−7.19% and 0.33%−0.26%, respectively. Other causes—including malignant skin melanoma, orofacial clefts, laryngeal cancer, and skin and head–neck cancers—each represented less than 1% of incident cases annually and showed minimal variation over time.

In terms of ASPR, pyoderma remained the leading contributor, with its share rising from 12.60 to 20.09% (Figure 3B). Breast cancer followed, increasing from 7.63 to 10.82%. Squamous-cell carcinoma showed a more than twofold rise (from 0.51 to 1.22%), while orofacial clefts (1.87%−2.42%) and malignant skin melanoma (0.67%−1.15%) also showed modest increases. Conversely, the proportion of Fire, heat, and hot substances injuries declined from 73.96 to 60.48% over the same period. Other conditions exhibited relatively stable contributions.

During the same period, the proportional composition of deaths attributable to plastic surgery–related diseases displayed distinct trends (Supplementary Figure S3A). Breast cancer remained the leading cause of death, increasing from 41.46 to 43.90%, followed by Lip and oral cavity cancer (11.11%−13.47%). The share of Fire, heat, and hot substances declined from 12.42 to 8.24%. Other conditions, including Pyoderma, orofacial clefts, and squamous-cell carcinoma, and Other pharynx cancer, each accounted for less than 5% of annual deaths. Regarding DALYs, breast cancer was the largest contributor throughout the period, increasing from 33.59 to 40.49% (Supplementary Figure S3B). Lip and oral cavity cancer (8.78%−11.47%) followed. The burden from Fire, heat, and hot substances injuries declined steadily (from 26.80 to 18.39%), while Larynx cancer remained stable (7.52%−6.06%). Nasopharynx cancer, and Other pharynx cancer jointly contributed around 10% of the total DALYs burden.

### Trends and correlations of plastic surgery-related diseases by socio-demographic index level

In 2021, incident cases were highest in low–middle SDI (301.21 million; 95% UI 291.53–311.51) and low SDI regions (253.33 million; 244.55–262.38), with the highest ASIR in low SDI settings, increasing from 22,279.24 in 1990 to 23,001.84 per 100,000 in 2021 [EAPC 0.11 (0.10–0.12); Table 1]. ASPR declined in all SDI groups, with the steepest reductions in high–middle SDI [from 2,995.25 in 1990 to 2,200.55 in 2021; EAPC −1.07 (−1.15 to −0.99)] and high SDI regions [from 4,390.32 to 3,177.34; −1.07 (−1.13 to −1.01); Supplementary Table S1]. Middle SDI regions recorded the highest number of deaths in 2021 [426,520 (375,790–477,629)], while low–middle SDI was the only group with an increasing ASDR [EAPC 0.17 (0.12–0.22); Supplementary Table S2]. Age-standardized DALYs rates declined across all quintiles, most notably in high–middle SDI [from 786.07 in 1990 to 490.66 in 2021; EAPC −1.83 (−1.94 to −1.72)] and high SDI regions [from 767.20 to 486.94; −1.51 (−1.53 to −1.48); Supplementary Table S3].

### Disease burden varied substantially by development level

**Figure 4:**
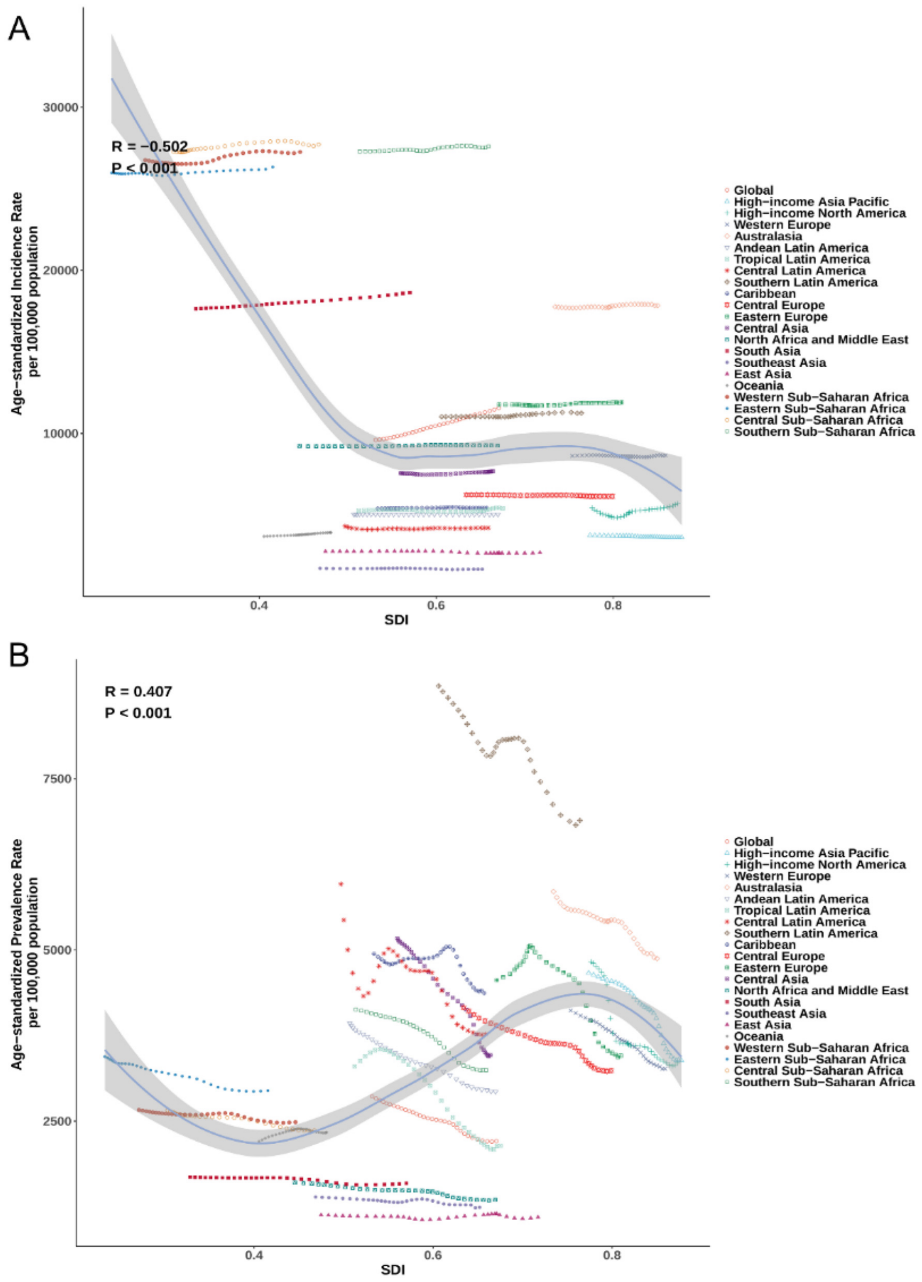
The **(A)** and ASPR **(B)** of plastic surgery-related diseases by 21 GBD regions and SDI, 1990–2021. ASIR, age-standardized incidence rate; ASPR, age-standardized prevalence rate; GBD, Global Burden of Disease; SDI, socio-demographic index.

SDI showed a moderate negative correlation with ASIR (*R* = −0.502; *P* < 0.001; Figure 4A): lower-SDI regions had higher incidence, with several exceeding 25,000 per 100,000, particularly in Central and Southern Sub-Saharan Africa. By contrast, SDI was positively correlated with ASPR (*R* = 0.407; *P* < 0.001; Figure 4B), with the highest prevalences clustering in high-SDI regions such as Southern Latin America and Australasia. For mortality, a weak but significant negative correlation was observed (*R* = −0.157; *P* < 0.001), with elevated ASDR concentrated in lower-SDI settings (Supplementary Figure S4A). Similarly, age-standardized DALYs rates declined with increasing SDI (*R* = −0.303; *P* < 0.001), with Southern Sub-Saharan Africa, Eastern Sub-Saharan Africa, and the Caribbean showing persistently high burdens despite some SDI improvement over time (Supplementary Figure S4B).

### Projected global burden of plastic surgery-related diseases by 2050

**Figure 5:**
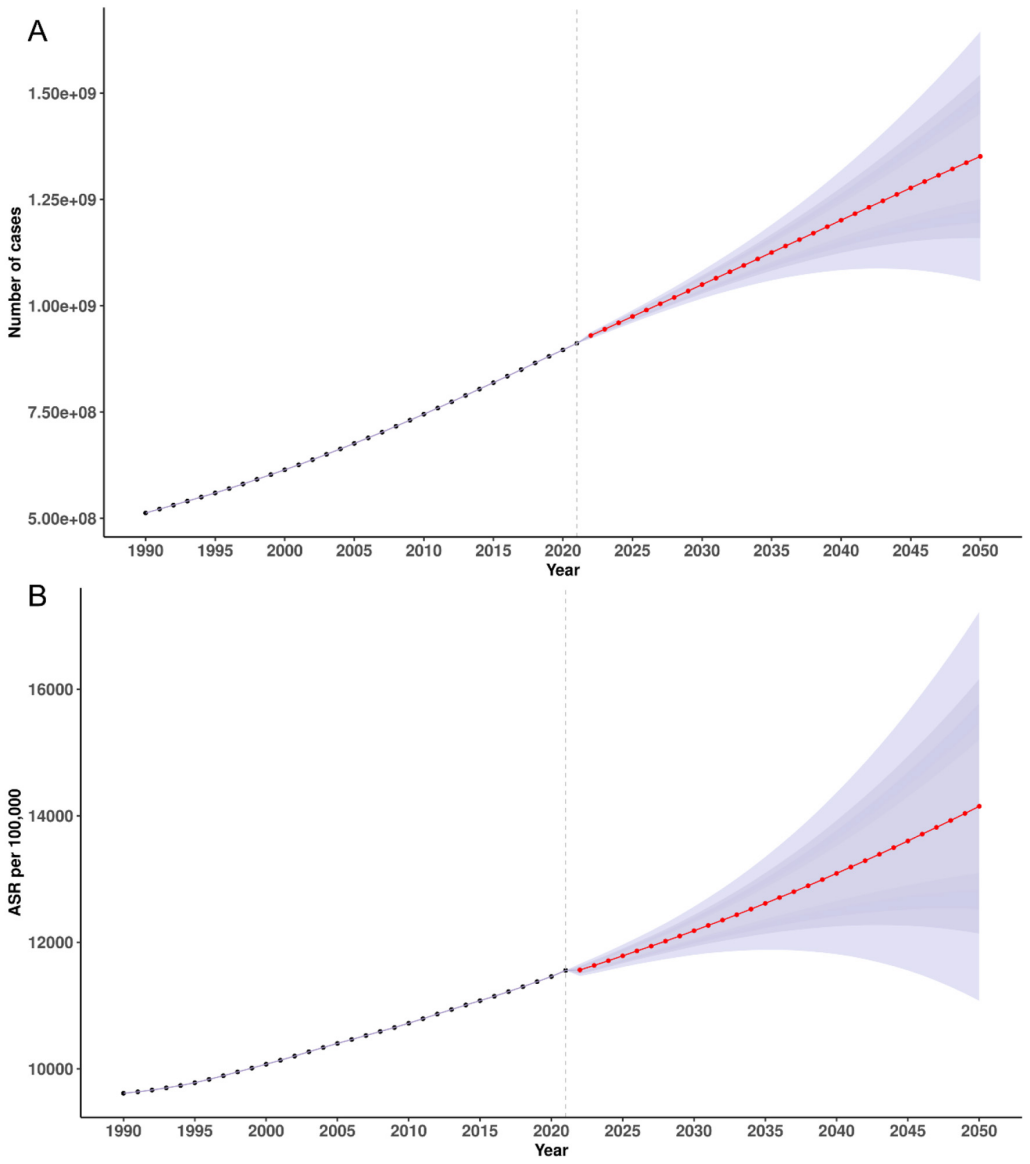
Temporal trends in incidence counts **(A)** and ASIR **(B)** for plastic surgery-related diseases, 1990–2021, with projections to 2050. ASIR, age-standardized incidence rate.

Based on BAPC projections, the global number of incident cases of plastic-surgery–related condition was expected to increase from 930.17 million (95% UI 922.35–937.99) in 2022 to 1,350.53 million (1,060.65–1,640.42) by 2050, a 45.2% rise (Figure 5A). Prevalent cases were projected to grow from 175.71 million (173.41–178.01) in 2022 to 193.68 million (167.26–220.11) by 2050 (Supplementary Figure S5A), a 10.2% increase. Deaths were forecast to decline from 1.45 million (1.43–1.48) in 2022 to 1.27 million (1.14–1.39) by 2050 (Supplementary Figure S6A), a 12.4% reduction. Similarly, DALYs were projected to fall from 46.83 million (46.07–47.59) in 2022 to 35.74 million (32.58–38.90) by 2050, a 23.7% decrease (Supplementary Figure S7A).

From a 2022 model-fitted baseline, the ASIR was expected to increase from 11,561.59 per 100,000 (95% UI 11,464.34–11,658.83) in 2022 to 14,139.48 (11,104.51–17,174.45) by 2050, a 22.3% rise (Figure 5B). By contrast, the ASPR was projected to decline slightly, from 2,184.00 (2,155.39–2,212.60) in 2022 to 2,027.79 (1,751.12–2,304.46) by 2050, a 7.2% reduction (Supplementary Figure S5B). The ASDR was forecast to decrease from 18.03 (17.72–18.34) in 2022 to 13.27 (11.97–14.57) by 2050, a 26.4% reduction (Supplementary Figure S6B). Similarly, the age-standardized DALYs rate was projected to decline from 582.07 (572.68–591.46) in 2022 to 374.19 (341.11–407.28) by 2050, a 35.7% decrease (Supplementary Figure S7B).

## Discussion

Our principal finding is that aggregate incidence is numerically dominated by pyoderma, whereas surgical service needs concentrate in oncologic, burn/trauma and congenital cohorts when assessed by deaths and DALYs. Accordingly, we interpret metrics by health-system relevance, anchoring surgical planning to deaths/DALYs and treating infection incidence as a primary-care and public-health signal (1, 2).

In practical terms, deaths and DALYs for breast, lip/oral cavity, naso-/laryngo-/other pharynx cancers, melanoma, non-melanoma skin cancer and burns indicate reconstructive capacity, peri-operative support (anesthesia/ICU) and rehabilitation needs; ASPR in oncologic and cleft cohorts informs survivorship follow-up, secondary reconstruction and scar/contracture management (18, 19); incidence of pyoderma/cellulitis chiefly signals primary-care and public-health workload (hygiene promotion, timely antibiotics, antimicrobial stewardship) with minimal implications for operative capacity (20); and burns incidence, interpreted alongside fatality and functional loss, informs emergency readiness, specialized burn units and rehabilitation.

At the regional level, the distribution of plastic surgery–related disease burden reveals persistent structural inequities in global surgical care. Regions such as South and East Asia, while demonstrating substantial absolute burdens, likely reflect a combination of demographic density and enhanced detection capacity driven by improved health information systems (11, 21). Conversely, the disproportionately high age-standardized rate in sub-Saharan Africa—particularly in its southern and western subregions—suggest a more complex interplay between delayed surgical access, limited health system readiness, and sustained exposure to preventable conditions such as trauma, infections, and advanced-stage cancers (1, 7, 22). The observed reductions in age-standardized death and disability rate in high-income regions point to the cumulative effect of decades-long investment in surgical infrastructure, early intervention pathways, and integrated rehabilitation services (2, 3, 23). However, the fact that some low- and middle-income regions continue to exhibit rising mortality and DALYs rate, despite epidemiological transition, underscores a critical need for context-specific strategies to strengthen surgical systems within broader health agendas (8, 24).

Surgical-relevant subgroups anchor service planning. Oncologic conditions (breast; lip/oral cavity, naso-/laryngo-/other pharynx; melanoma; non-melanoma skin cancer) require coordinated resection–reconstruction pathways and survivorship rehabilitation (25). Burns require regionalized acute care, grafting capacity, and long-term contracture release with rehabilitation (26). Orofacial clefts call for integrated programmes (primary repair, speech/orthodontic care) and backlog reduction (19). Infections (pyoderma, cellulitis) should be decoupled from surgical capacity planning and addressed through community prevention and early case management; surgical input is episodic (e.g., abscess drainage) rather than capacity-defining (20). These choices are consistent with cause-specific burden patterns in GBD 2021 (13).

At the national level, the heterogeneity in burden patterns reflects not only variations in demographic structure and population size, but also the uneven distribution of surgical capacity and political prioritization of reconstructive care (2, 8). Countries with large populations, such as India and China, contribute substantially to the global case burden, but differences in standardized rate between nations with similar socioeconomic profiles highlight the influence of national health policies, coverage schemes, and disease surveillance quality (21). The persistence of high incidence and mortality in several smaller or middle-income countries may also be driven by localized environmental risks, injury burden, or under-resourced referral networks (23, 27). Striking reductions in burden observed in selected nations indicate that even within constrained settings, targeted interventions—such as task-shifting, subsidized surgical programs, or community-based early detection—can yield measurable improvements (22, 28). These national disparities emphasize the importance of tailoring global surgical development efforts to country-specific contexts, ensuring that progress is not only broad but equitably distributed.

Age-specific patterns in the burden of plastic surgery–related diseases underscore the dual vulnerability of the very young and the very old, reflecting distinct biological, clinical, and health system challenges across the life course (6, 29). The observed U-shaped distribution of incidence, with elevated rate among young children and older adults, may be driven by congenital anomalies and infection-related conditions in early life, and by degenerative, oncological, or pressure-related pathologies in later years (30). The steep gradient in prevalence, mortality, and DALYs rate with advancing age likely reflects not only the cumulative burden of comorbidities and frailty, but also disparities in surgical access, rehabilitation capacity, and complication management in aging populations (2, 31). Despite modest declines in age-specific mortality and DALYs rate across many age groups—likely attributable to improvements in perioperative care and earlier intervention—the persistently high burden among individuals aged 80 years and older signals unmet needs in geriatric surgical care (32). Furthermore, the growing absolute burden in this age group points to the demographic reality of global population aging and its implications for reconstructive surgical demand. These findings highlight the urgency of integrating age-sensitive surgical planning into broader health system design, including investment in workforce training, long-term care pathways, and multidisciplinary models suited to older adults (33).

The evolving cause-specific composition of plastic surgery–related disease burden reflects shifting epidemiological profiles, sociocultural determinants, and differential access to preventive and surgical care. The dominance of pyoderma as the leading cause of incidence underscores the persistent global impact of skin infections, particularly in tropical and low-resource settings where overcrowding, poor hygiene, and limited access to primary care amplify transmission risks (34, 35). However, the disproportionately high contribution of pyoderma—accounting for over 90% of incident cases throughout the study period—warrants careful interpretation. This pattern may reflect the broad case definition of pyoderma within the GBD framework, which aggregates a wide spectrum of superficial and deep bacterial skin infections (33). Given the ubiquity and recurrent nature of these conditions, especially in vulnerable environments, their high incidence estimates may partially reflect repeated episodes in the same individuals or limited diagnostic specificity in primary data sources (36). While such infections are clinically relevant, their numerical dominance may obscure the relative impact of more resource-intensive surgical conditions such as breast cancer, congenital anomalies, and thermal injuries. This imbalance has important implications for surgical planning and policy, as it may distort prioritization and resource allocation within reconstructive health systems. Refinement of case definitions and disaggregation by severity would be valuable in future burden assessments to ensure more accurate alignment with clinical need and system capacity (12, 37).

Meanwhile, the growing contributions of breast cancer and squamous-cell carcinoma to both prevalence and DALYs suggest improved case detection and survival, as well as expanding indications for reconstructive surgery following oncological treatment (38). The rising share of orofacial clefts may reflect enhancements in diagnostic capacity and registry reporting, yet also signals an unmet need for timely surgical correction and post-operative support in many settings (33). By contrast, the declining proportional burden of injuries caused by fire, heat, and hot substances likely results from improved injury prevention, regulation, and emergency response systems—though these conditions still constitute a major source of morbidity in certain regions (39). The consistent predominance of breast and head–neck malignancies in death and DALYs patterns highlights the long-term functional and psychosocial sequelae of these diseases, and underscores the importance of integrating plastic and reconstructive surgery into comprehensive cancer care frameworks (38, 40). Collectively, these trends emphasize the need for cause-targeted investment in surgical capacity, rehabilitation services, and upstream public health interventions tailored to regional disease profiles.

Marked gradients by SDI underscore structural inequities in access, quality, and broader social determinants. In low- and low-middle SDI settings, persistent exposures (untreated infections, trauma, perinatal conditions), limited early intervention, and fragile referral/workforce capacity drive higher incidence and late presentation, increasing the need for complex reconstruction (28, 41). By contrast, in high-SDI settings, ASIR is generally lower but ASPR can be comparable or higher—consistent with improved survival and longer duration of living with disease following earlier diagnosis and treatment—while ASPR/ASDR decline more steeply, reflecting broader coverage of effective interventions, peri-operative care, and rehabilitation. Notably, a small rise in ASIR in high-income North America likely reflects earlier detection, coding completeness, and survivorship dynamics rather than worsening underlying risk. These patterns are coherent with differences in health-system capacity and effective coverage and are amplified by population aging, which raises prevalent caseloads even as rates fall; persistent residual burden in regions such as sub-Saharan Africa and parts of the Caribbean highlights the need to embed essential and reconstructive surgical services within UHC and tailor delivery to regional capacities (24, 42).

Projections to 2050 reveal a divergent pattern in the global burden of conditions relevant to reconstructive care: while age-standardized mortality and DALYs rates are expected to continue declining, the absolute number of cases is projected to rise. This contrast largely reflects demographic inertia—population growth and aging—particularly in low- and middle-income settings (43, 44). Beyond demography, increases in aggregate incidence are chiefly infection-driven (notably pyoderma), with more limited contributions from diagnostic capacity, case definitions, and surveillance in selected subgroups (e.g., oncologic and some congenital/chronic wound conditions) (39, 45). For pyoderma, demographic expansion and environmental exposures (sanitation, hygiene, crowding, timely antibiotics) are more plausible drivers than diagnostic shifts; by contrast, improved detection and evolving practice may partly raise oncologic case ascertainment. Against this backdrop, surgically relevant pressures are better captured by deaths and DALYs within oncologic, burn/trauma, and congenital subgroups, whose trajectories diverge by SDI; service planning should therefore be anchored to these DALYs trends rather than to incidence.

In parallel, the observed decline in age-standardized mortality and DALYs rates suggests substantial improvements in surgical care delivery. Advances in perioperative management, anesthesia safety, and infection control, along with expanded access to rehabilitation services, have collectively improved survival and reduced long-term disability after surgery (23, 46). These relative gains, however, may not be sufficient to offset the surge in demand generated by population aging and shifting disease profiles. Without strategic health system planning, this growing mismatch between surgical need and capacity may exacerbate existing inequities (42). The projected rise in absolute burden underscores the urgent necessity of integrating essential surgical services into universal health coverage frameworks and expanding investment in workforce development, surgical infrastructure, and post-operative care—particularly in regions where demographic and epidemiologic transitions are most rapid (38, 47).

This study provides the first comprehensive global analysis of the burden of plastic surgery–related diseases using data from the Global Burden of Disease Study 2021, covering 204 countries and territories over a 30-year period. By incorporating multiple standardized burden indicators—including incidence, prevalence, mortality, and DALYs—the study offers a multidimensional perspective on epidemiological trends. The use of age-period-cohort modeling enables robust long-term projections, highlighting future demands for surgical services under population aging and epidemiological transition. In addition, the analysis of SDI gradients and cause-specific burden reveals structural disparities, informing health policy prioritization.

However, several limitations warrant emphasis. First-and centrally-the included conditions are heterogeneous, so aggregate incidence is infection-dominated while surgical demand lies in oncologic, burn/trauma, and congenital subgroups; we therefore treat composites descriptively and anchor service implications to deaths/DALYs, recognizing residual issues from broad pyoderma coding and limited trauma granularity. Second, reliance on GBD 2021 secondary data may introduce bias in data-sparse settings. Third, BAPC projections cannot anticipate disruptive shocks (conflict, pandemics, and policy shifts). Future work should add procedure-level/claims data, finer coding, and multisource validation to improve the surgical signal.

## Conclusion

The burden of conditions relevant to reconstructive care remains substantial and heterogeneous across ages, regions, and SDI strata. While age-standardized mortality and DALYs rates declined, absolute cases rose with population growth and aging. Aggregate incidence is dominated by infections (notably pyoderma)—a primary-care/public-health signal—whereas surgically relevant burden concentrates in breast and head-and-neck cancers, burns, and selected congenital anomalies. Accordingly, incidence should inform prevention and community management, whereas surgical capacity planning should be anchored to deaths/DALYs in oncologic, burn/trauma, and congenital cohorts. Looking to 2050, infection-driven incidence is projected to keep rising, with DALYs trajectories diverging by SDI, underscoring priorities of context-specific prevention, timely cancer detection and treatment, regionalised burn care, comprehensive cleft programmes, and integrated rehabilitation. Embedding essential surgical and reconstructive services within universal health coverage—through workforce development, peri-operative infrastructure, and sustainable financing—will be pivotal to improving equity and functional outcomes.

## Data Availability

The original contributions presented in the study are included in the article/Supplementary material, further inquiries can be directed to the corresponding author.

## References

[1] Meara JG Leather AJ Hagander L Alkire BC Alonso N Ameh EA . Global Surgery 2030: evidence and solutions for achieving health, welfare, and economic development. Am J Obstet Gynecol. (2015) 213:338–40. doi: 10.1016/j.surg.2015.02.009 25985722

[2] Debas HT Donkor P Gawande A Jamison DT Kruk ME Mock CN. Essential Surgery: Disease Control Priorities. 3rd ed, Vol 1. Washington, DC: World Bank (2015). 26740991

[3] World Health Organization. Global Priorities for Surgical Care: Strengthening Public Health through Surgery. Geneva, Switzerland: WHO (2021).

[4] Henry JA Frenkel E Borgstein E Mkandawire N Goddia C. Surgical and anaesthetic capacity of hospitals in Malawi: key insights. Health Policy Plan. (2015) 30:985–94. doi: 10.1093/heapol/czu102 25261799 PMC4559113

[5] Shrime MG Dare AJ Alkire BC O'Neill K Meara JG. Catastrophic expenditure to pay for surgery worldwide: a modelling study. Lancet Glob Health. (2015) 3:S38–44. doi: 10.1016/S2214-109X(15)70085-9 25926319 PMC4428601

[6] Ma Q Wei J Peng B Liu J Mo S. Burden of orofacial clefts from 1990-2021 at global, regional, and national levels. Front Pediatr. (2025) 13:1502877. doi: 10.3389/fped.2025.1502877 40191646 PMC11968431

[7] Shrime MG Bickler SW Alkire BC Mock C. Global burden of surgical disease: an estimation from the provider perspective. Lancet Glob Health. (2015) 3(Suppl. 2):S8–9. doi: 10.1016/S2214-109X(14)70384-5 25926322

[8] Kebede MA Tor DSG Aklilu T Petros A Ifeanyichi M Aderaw E . Identifying critical gaps in research to advance global surgery by 2030: a systematic mapping review. BMC Health Serv Res. (2023) 23:946. doi: 10.1186/s12913-023-09973-9 37667225 PMC10478287

[9] Raykar NP Ng-Kamstra JS Bickler S Davies J Greenberg SLM Hagander L . New global surgical and anaesthesia indicators in the World Development Indicators dataset. BMJ Glob Health. (2017) 2:e000265. doi: 10.1136/bmjgh-2016-000265 29225929 PMC5717956

[10] Wang H Abbas KM Abbasifard M Abbasi-Kangevari M Abedi A Abreu DMX . Global age-sex-specific fertility, mortality, healthy life expectancy, and population estimates and projections, 1990–2100: a forecasting analysis for the GBD Study. Lancet. (2020) 396:1160–203. doi: 10.1016/S0140-6736(20)30977-6 33069325 PMC7566045

[11] GBD 2021 Diseases and Injuries Collaborators. Global incidence, prevalence, YLDs, DALYs, and HALE for 371 diseases and injuries in 204 countries and territories and 811 subnational locations, 1990-2021: a systematic analysis for the Global Burden of Disease Study 2021. Lancet. (2024) 403:2133–61. doi: 10.1016/S0140-6736(24)00757-8 38642570 PMC11122111

[12] James SL Abate D Abate KH Abay SM Abbafati C Abbasi N . Global, regional, and national incidence, prevalence, and years lived with disability for 354 diseases and injuries, 1990–2017. Lancet. (2018) 392:1789–858. doi: 10.1016/S0140-6736(18)32279 30496104 PMC6227754

[13] Rue H Martino S Chopin N. Approximate Bayesian inference for latent Gaussian models using INLA. J R Stat Soc B. (2009) 71:319–92. doi: 10.1111/j.1467-9868.2008.00700.x

[14] GBD 2021 Causes of Death Collaborators. Global burden of 288 causes of death and life expectancy decomposition in 204 countries and territories and 811 subnational locations, 1990-2021: a systematic analysis for the Global Burden of Disease Study 2021. Lancet. (2024) 403:2100–32. doi: 10.1016/S0140-6736(24)00367-2 38582094 PMC11126520

[15] IHME GHDx. Global Burden of Disease Study 2021 (GBD 2021) Cause–ICD Code Mappings (ICD-9/ICD-10 Crosswalks for GBD causes). Seattle, WA: Institute for Health Metrics and Evaluation (2024).

[16] Sardiwalla Y Price EL Bridgman AC Voineskos S. The burden of plastic surgery–related disease in Canada: a perspective based on the 2019 Global Burden of Disease Study. Plast Surg. (2024) 32:481–9. doi: 10.1177/22925503221108447 39104942 PMC11298130

[17] Riebler A Held L. Projecting the future burden of cancer: Bayesian age-period-cohort analysis with integrated nested Laplace approximations. Biom J. (2017) 59:531–49. doi: 10.1002/bimj.201500263 28139001

[18] Ranganathan K Singh P Raghavendran K Wilkins EG Hamill JB Aliu O . The Global macroeconomic burden of breast cancer: implications for oncologic surgery. Ann Surg. (2021) 274:1067–72. doi: 10.1097/SLA.0000000000003662 32097168

[19] Massenburg BB Hopper RA Crowe CS Morrison SD Alonso N Calis M . Global burden of orofacial clefts and the world surgical workforce. Plast Reconstr Surg. (2021) 148:568e−80e. doi: 10.1097/PRS.0000000000008334 34550940

[20] Stevens DL Bisno AL Chambers HF Dellinger EP Goldstein EJ Gorbach SL . Practice guidelines for the diagnosis and management of skin and soft tissue infections: 2014 update by the Infectious Diseases Society of America. Clin Infect Dis. (2014) 59:e10–52. doi: 10.1093/cid/ciu296 24973422

[21] Boerma T Eozenou P Evans D Evans T Kieny MP Wagstaff A. Monitoring progress towards universal health coverage at country and global levels. PLoS Med. (2014) 11:e1001731. doi: 10.1371/journal.pmed.1001731 25243899 PMC4171369

[22] Ng-Kamstra JS Greenberg SLM Abdullah F Amado V Anderson GA Cossa M . Global Surgery 2030: a roadmap for high income country actors. BMJ Glob Health. (2016) 1:e000011. doi: 10.1136/bmjgh-2015-000011 28588908 PMC5321301

[23] Grimes CE Bowman KG Dodgion CM Lavy CB. Systematic review of barriers to surgical care in low-income and middle-income countries. World J Surg. (2011) 35:941–50. doi: 10.1007/s00268-011-1010-1 21360305

[24] Morton DG Ghaffar A. Strengthening health systems through surgery. BMJ Glob Health. (2024) 9(Suppl 4):e017782. doi: 10.1136/bmjgh-2024-017782 39510563 PMC11575301

[25] Sullivan R Alatise OI Anderson BO Audisio R Autier P Aggarwal A . Global cancer surgery: delivering safe, affordable, and timely cancer surgery. Lancet Oncol. (2015) 16:1193–224. doi: 10.1016/S1470-2045(15)00223-5 26427363

[26] Jeschke MG van Baar ME Choudhry MA Chung KK Gibran NS Logsetty S. Burn injury. Nat Rev Dis Primers. (2020) 6:11. doi: 10.1038/s41572-020-0145-5 32054846 PMC7224101

[27] Peters DH Garg A Bloom G Walker DG Brieger WR Hafizur Rahman M. Poverty and access to health care in developing countries. Ann N Y Acad Sci. (2008) 1136:161–71. doi: 10.1196/annals.1425.011 17954679

[28] Citron I Chokotho L Lavy C. Prioritisation of surgery in the National Health Strategic Plans of Africa: a systematic review. World J Surg. (2016) 40:779–83. doi: 10.1007/s00268-015-3333-9 26711637 PMC4767853

[29] Prince MJ Wu F Guo Y Gutierrez-Robledo LM O'Donnell M Sullivan R . The burden of disease in older people and implications for health policy and practice. Lancet. (2015) 385:549–62. doi: 10.1016/S0140-6736(14)61347-7 25468153

[30] Zhou M Wang H Zhu J Chen W Wang L Liu S . Cause-specific mortality for 240 causes in China during 1990–2013: a systematic subnational analysis for the Global Burden of Disease Study 2013. Lancet. (2016) 387:251–72. doi: 10.1016/S0140-6736(15)00551-6 26510778

[31] Dindo D Demartines N Clavien P-A. Classification of surgical complications: a new proposal with evaluation in a cohort of 6336 patients and results of a survey. Ann Surg. (2004) 240:205–13. doi: 10.1097/01.sla.0000133083.54934.ae 15273542 PMC1360123

[32] Alrezk R Jackson N Al Rezk M Elashoff R Weintraub N Elashoff D . Derivation and validation of a geriatric-sensitive perioperative cardiac risk index. J Am Heart Assoc. (2017) 6:e006648. doi: 10.1161/JAHA.117.006648 29146612 PMC5721761

[33] Howlader N Noone AM Krapcho M Miller D Brest A Yu M ., editors. SEER Cancer Statistics Review, 1975–2018. Bethesda, MD: National Cancer Institute (2021).

[34] Hay RJ Johns NE Williams HC Bolliger IW Dellavalle RP Margolis DJ . The global burden of skin disease in 2010: an analysis of the prevalence and impact of skin conditions. J Invest Dermatol. (2014) 134:1527–34. doi: 10.1038/jid.2013.446 24166134

[35] Karimkhani C Dellavalle RP Coffeng LE Flohr C Hay RJ Langan SM . Global skin disease morbidity and mortality: an update from the Global Burden of Disease Study 2013. JAMA Dermatol. (2017) 153:406–12. doi: 10.1001/jamadermatol.2016.5538 28249066 PMC5817488

[36] Walker SL Shah M Hubbard VG Pradhan HM Ghimire M. Skin disease is common in rural Nepal: results of a point prevalence study. Br J Dermatol. (2008) 158:334–8. doi: 10.1111/j.1365-2133.2007.08107.x 17711533

[37] Mock CN Donkor P Gawande A Jamison DT Kruk ME Debas HT. Essential surgery: key messages from Disease Control Priorities, 3rd edition. Lancet. (2015) 385:2209–19. doi: 10.1016/S0140-6736(15)60091-5 25662414 PMC7004823

[38] Cordeiro PG. Breast reconstruction after surgery for breast cancer. N Engl J Med. (2008) 359:1590–601. doi: 10.1056/NEJMct0802899 18843123

[39] Peck MD. Epidemiology of burns throughout the world. Part I: Distribution and risk factors. Burns. (2011) 37:1087–100. doi: 10.1016/j.burns.2011.06.005 21802856

[40] Krouse RS Grant M Ferrell B Dean GE Nelson RA Chu DZ. Quality of life outcomes in 599 cancer patients undergoing ostomy surgery. Psychooncology. (2007) 16:795–804. doi: 10.1016/j.jss.2006.04.033 17196990

[41] Wilkinson E Aruparayil N Gnanaraj J Brown J Jayne D. Barriers to training in laparoscopic surgery in low- and middle-income countries: a systematic review. Trop Doct. (2021) 51:408–14. doi: 10.1177/0049475521998186 33847545 PMC8411480

[42] Alkire BC Raykar NP Shrime MG Weiser TG Bickler SW Rose JA . Global access to surgical care: a modelling study. Lancet Glob Health. (2015) 3:e316–23. doi: 10.1016/S2214-109X(15)70115-4 25926087 PMC4820251

[43] Beard JR Officer A de Carvalho IA Sadana R Pot AM Michel JP . The World report on ageing and health: a policy framework for healthy ageing. Lancet. (2016) 387:2145–54. doi: 10.1016/S0140-6736(15)00516-4 26520231 PMC4848186

[44] GBD 2019 Diseases and Injuries Collaborators. Global burden of 369 diseases and injuries in 204 countries and territories, 1990–2019: a systematic analysis for the Global Burden of Disease Study 2019. Lancet. (2020) 396:1204–22. doi: 10.1016/S0140-6736(20)30925-9 33069326 PMC7567026

[45] Yoon AP Qi J Kim HM Hamill JB Jagsi R Pusic AL . Patient-reported outcomes after irradiation of tissue expander versus permanent implant in breast reconstruction: a multicenter prospective study. Plast Reconstr Surg. (2020) 145:917e−26e. doi: 10.1097/PRS.0000000000006724 32332528 PMC7184969

[46] Weiser TG Haynes AB Molina G Lipsitz SR Esquivel MM Uribe-Leitz T . Estimate of the global volume of surgery in 2012: an assessment supporting improved health outcomes. Lancet. (2015) 385:S11. doi: 10.1016/S0140-6736(15)60806-6 26313057

[47] Alkire BC Raykar NP Shrime MG Weiser TG Bickler SW Rose JA Nutt CT Farmer PE Meara JG. Health system strengthening and surgery: building resilience and equity through integration. World J Surg. (2015) 39:2111–7. doi: 10.1007/s00268-015-3153-126178660

