# Supplementary figures and images for: Global burden of plastic-surgery-related conditions, 1990–2021: a composition-aware analysis with projections to 2050
Source: Front Public Health. 2025 Nov 12;13:1676386. doi: 10.3389/fpubh.2025.1676386 (PMC12647102; doi:10.3389/fpubh.2025.1676386)

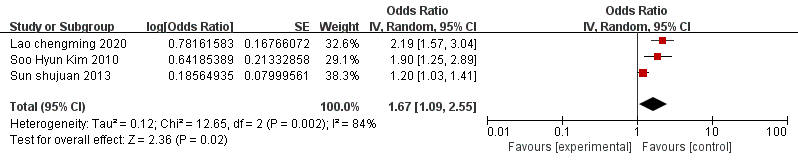

Supplement: Supplementary file 1 [file Data_Sheet_1.ZIP › Capical figure/Capical figure/age.png]

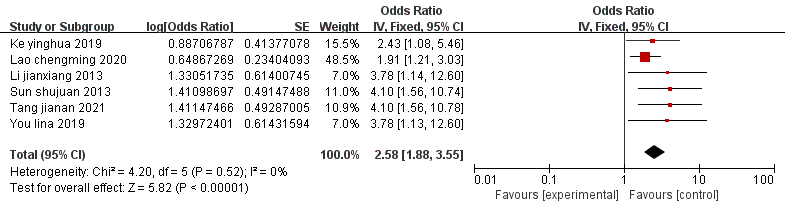

Supplement: Supplementary file 1 [file Data_Sheet_1.ZIP › Capical figure/Capical figure/disease cognition.png]

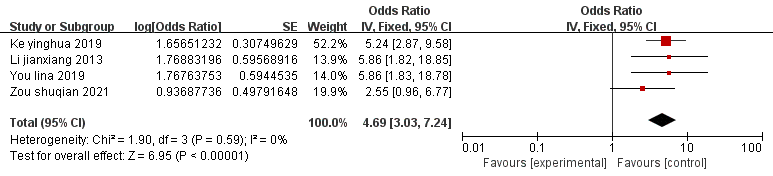

Supplement: Supplementary file 1 [file Data_Sheet_1.ZIP › Capical figure/Capical figure/hysterectomy.png]

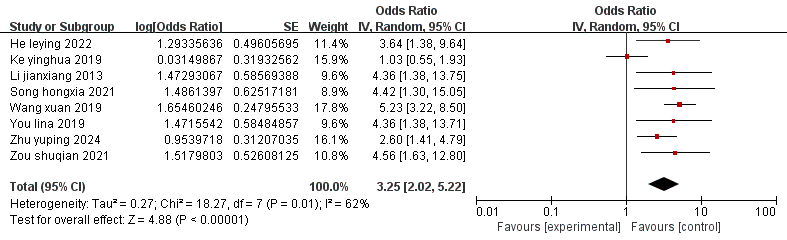

Supplement: Supplementary file 1 [file Data_Sheet_1.ZIP › Capical figure/Capical figure/low education level.png]

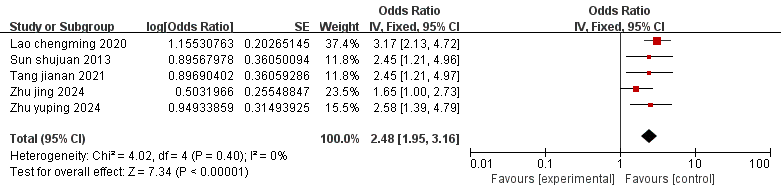

Supplement: Supplementary file 1 [file Data_Sheet_1.ZIP › Capical figure/Capical figure/Low social support.png]

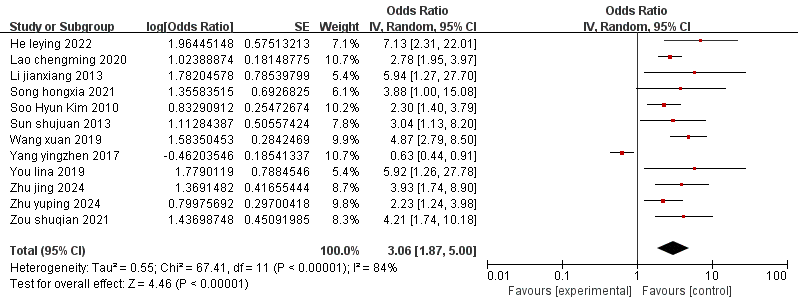

Supplement: Supplementary file 1 [file Data_Sheet_1.ZIP › Capical figure/Capical figure/monthly income.png]

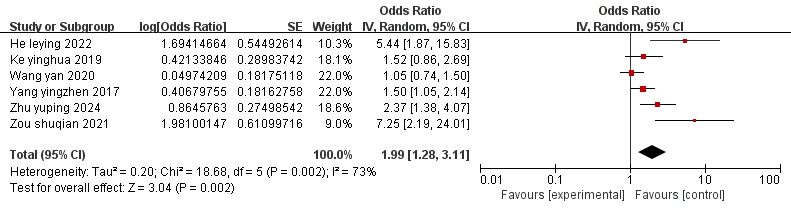

Supplement: Supplementary file 1 [file Data_Sheet_1.ZIP › Capical figure/Capical figure/neoplasm staging.png]

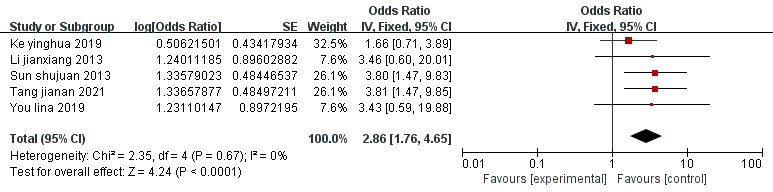

Supplement: Supplementary file 1 [file Data_Sheet_1.ZIP › Capical figure/Capical figure/Pain.png]

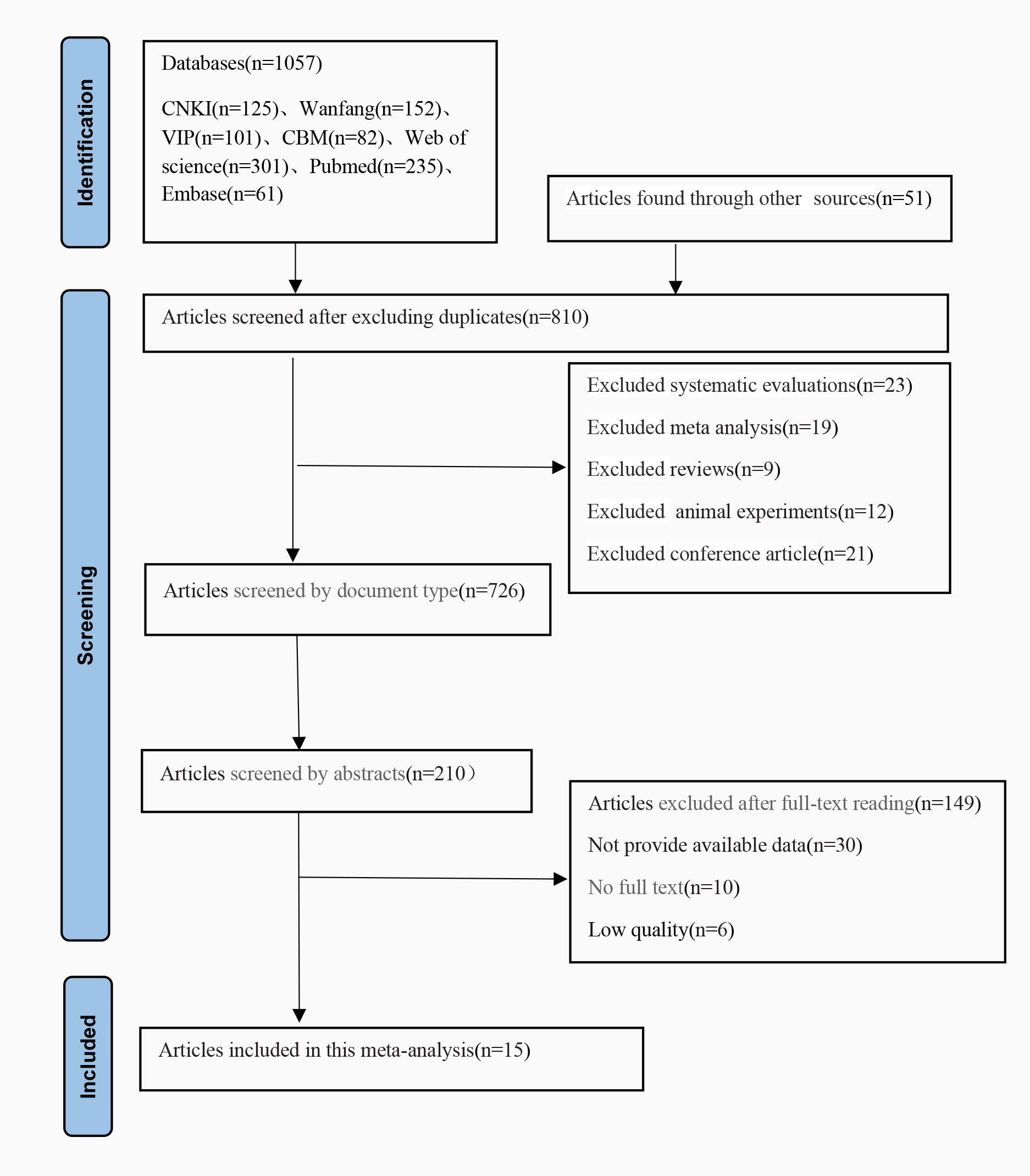

Supplement: Supplementary file 1 [file Data_Sheet_1.ZIP › Capical figure/Literature screening flowchart.tif]
